# Genetic variability and history of a native Finnish horse breed

**DOI:** 10.1186/s12711-019-0480-8

**Published:** 2019-07-01

**Authors:** Laura Kvist, Markku Niskanen, Kristiina Mannermaa, Saskia Wutke, Jouni Aspi

**Affiliations:** 1 Department of Ecology and Genetics, University of Oulu, POB 8000, 90014 Oulu, Finland; 2 Research Unit of History, Culture and Communications, University of Oulu, POB 8000, 90014 Oulu, Finland; 3 Department of Philosophy, History, Culture and Art Studies, University of Helsinki, POB 24, 00014 Helsinki, Finland; 4 Department of Environmental and Biological Sciences, University of Eastern Finland, POB 111, 80101 Joensuu, Finland

## Abstract

**Background:**

The Finnhorse was established as a breed more than 110 years ago by combining local Finnish landraces. Since its foundation, the breed has experienced both strong directional selection, especially for size and colour, and severe population bottlenecks that are connected with its initial foundation and subsequent changes in agricultural and forestry practices. Here, we used sequences of the mitochondrial control region and genomic single nucleotide polymorphisms (SNPs) to estimate the genetic diversity and differentiation of the four Finnhorse breeding sections: trotters, pony-sized horses, draught horses and riding horses. Furthermore, we estimated inbreeding and effective population sizes over time to infer the history of this breed.

**Results:**

We found a high level of mitochondrial genetic variation and identified 16 of the 18 haplogroups described in present-day horses. Interestingly, one of these detected haplogroups was previously reported only in the Przewalski’s horse. Female effective population sizes were in the thousands, but declines were evident at the times when the breed and its breeding sections were founded. By contrast, nuclear variation and effective population sizes were small (approximately 50). Nevertheless, inbreeding in Finnhorses was lower than in many other horse breeds. Based on nuclear SNP data, genetic differentiation among the four breeding sections was strongest between the draught horses and the three other sections (*F*_ST_ = 0.007–0.018), whereas based on mitochondrial DNA data, it was strongest between the trotters and the pony-sized and riding horses (Φ_ST_ = 0.054–0.068).

**Conclusions:**

The existence of a Przewalski’s horse haplogroup in the Finnhorse provides new insights into the domestication of the horse, and this finding supports previous suggestions of a close relationship between the Finnhorse and eastern primitive breeds. The high level of mitochondrial DNA variation in the Finnhorse supports its domestication from a large number of mares but also reflects that its founding depended on many local landraces. Although inbreeding in Finnhorses was lower than in many other horse breeds, the small nuclear effective population sizes of each of its breeding sections can be considered as a warning sign, which warrants changes in breeding practices.

**Electronic supplementary material:**

The online version of this article (10.1186/s12711-019-0480-8) contains supplementary material, which is available to authorized users.

## Background

Domestication of animals and plants has played an essential role in human history. At present, there are more than 8800 breeds of 38 domesticated animal species listed in the Domestic Animal Diversity Information System (DAD-IS) [1]. Domestication has influenced the behaviour, morphology, physiology and performance of a species through selective breeding. It has also led to the fixation of breed-specific traits through inbreeding, genetic drift, founder effect and selection [2, 3]. Two examples of the occasionally large phenotypic differences between wild and domesticated animals are the great variation in coat colour [4] and body size (e.g., in dogs; see, for example, [5]) in domesticated species that still often show limited variation within breeds. Thus, captive breeding may decrease the overall genetic variation but may increase it for certain traits.

A study of the genome-wide genetic diversity of 36 horse breeds [6] based on single nucleotide polymorphism (SNP) genotyping data showed that the diversity was low in breeds that have experienced high selective pressures, been closed populations, gone through a population bottleneck, or have low current census sizes (e.g., the Exmoor, Clydesdale and Florida Cracker breeds, with expected heterozygosity H_E_ ranging from 0.236 to 0.291). By contrast, a high level of diversity was detected in landraces or breeds that were old, had large population sizes, were outcrossing, displayed a high level of phenotypic diversity, or had experienced weaker artificial selection (e.g., Mongolian, Tuva and New Forest Pony; H_E_ ranging from 0.314 to 0.322). The extremely low genetic variation of the horse Y-chromosome demonstrates that the domestic horse has only few paternal founders [7–9], whereas the genetic variation of the maternally-inherited mitochondrial DNA is very high [10–12].

In this study, we examined the genomic and mitochondrial diversity of the Finnhorse, which is an all-round horse breed and the national horse breed of Finland. It is closely related to other Nordic or eastern horse breeds, e.g., the Norwegian Fjord, North Swedish horse, Gotland Russ, Icelandic horse, Estonian horse and Mongolian horse [6, 13]. Historical sources have revealed the existence of several breeding programs since, at least, the sixteenth century, which aimed mostly at increasing the size of the Finnish horse by crossing it with larger European breeds [14]. The history of this breed since the foundation of the studbook in 1907 is well recorded [15]. The number of Finnhorses declined strongly during the urbanization of Finland in the 1960s and 1970s, when people moved from rural areas into cities and agricultural and forestry practice began to use motorized ‘horsepower’ instead of real horses. The number of Finnhorses declined from over 400,000 in the 1950s to an all-time small number of 14,000 in 1987 [16], later increasing to the present number of approximately 20,000. Finnhorses are currently bred in four breeding sections: harness trotters (since 1965; 218 stallions and 1575 mares in 2017), riding horses (since 1971; 103 stallions and 611 mares), pony-sized horses (since 1971; 64 stallions and 219 mares) and draught horses (since 1971; 30 stallions and 103 mares [15]). To be accepted in the studbook, the horses of each section need to fill certain section-specific criteria that include size, conformation, performance, gaits, health and behaviour. In practice, most Finnhorses are not evaluated before entering the studbook, but for an individual to be registered as a Finnhorse, it must trace back at least three generations of Finnhorses [15].

At the beginning of the twentieth century, when the studbook was founded, Finnhorses were strongly selected for colour (chestnut) and size (at least 148 cm at the withers), and they were bred first for draught purposes to work in agriculture and forestry and, later also as a lighter, all-round cavalry horse [17]. Selection was subsequently directed towards traits that were specific to each breeding section (e.g., maximum withers height of 148 cm for pony-sized horses, pulling capability for draught horses, athletic conformation for harness trotters and rhythmic, elastic and advancing gaits for riding horses [15, 17]). It is assumed that while all present-day Finnhorses can be traced back to only four founding stallions, which were born between 1879 and 1929, the number of founding mares was large [17]. The small number of founding stallions, together with the strict breeding standards, presumably decreased the genetic variation of the breed at the beginning of the 1900s. However, the later formation of the four separate breeding sections likely increased genetic differentiation. In addition, the decline in numbers until the 1980s created a population bottleneck, which possibly further decreased the genetic diversity and increased the effect of genetic drift. Today, the number of individuals in the draught horse breeding section, in particular, is alarmingly small. However, according to [6], the levels of heterozygosity and inbreeding in the Finnhorse breed are not markedly different from those of other horse breeds (H_E_ = 0.301, inbreeding coefficient F_IS_ = − 0.004 and effective population size N_e_ = 575 estimated from 27 Finnhorses, whereas the corresponding mean values for all studied breeds are equal to 0.295, 0.007 and 341, respectively). This higher than expected level of heterozygosity and lower than expected inbreeding coefficient for a breed with strong directional selection and a recent decrease in population size have been explained by its within-breed genetic structure [6], i.e. its differentiation into four breeding sections.

It is still unknown how the history of the Finnhorse has influenced the genetic diversity of the breed as a whole or the differentiation among its four breeding sections. Therefore, our aims were to (1) characterize the genetic variation of the breed and breeding sections by analyses based on whole-genome SNPs and mitochondrial DNA sequences, (2) estimate the genomic and mitochondrial differentiation between the four breeding sections, and (3) estimate inbreeding and effective population sizes.

## Methods

### Sampling and DNA extraction

Nine hundred and ninety-one samples of horses were obtained either directly from horse owners (hair samples from the mane or tail, N = 960) or from a horse hospital (blood samples, with the permission from the owners, N = 31). Among these, 852 samples were from Finnhorses (67 harness trotters, 79 riding horses, 30 draught horses, 51 pony-sized horses and 641 individuals that are not registered in the studbook) with 16 individuals that were concurrently registered in two breeding sections (three as trotter and riding, three as riding and draught, five as riding and pony-sized, one as draught and pony-sized, four as trotter and draught), and thus they were included in both breeding sections for the estimations of genetic diversity and differentiation between sections. We also analysed samples from several other breeds: eight horses from different Baltic breeds (two Estonian horses, one Estonian riding pony, two Estonian Sport horses, one Tori horse, two Latvian Sport horses and one horse of unknown breed that originated from Estonia), 30 Warmblood Trotters (including seven American Trotters), seven Finnish Warmbloods (FWB), two Hanoverian horses, two Royal Dutch Sport horses (KWPN), three Warmblood Riding horses, one Oldenburg horse, one Zangersheide horse, one American Quarter horse, one Friesian horse, two Irish Cobs, three Welsh Mountain ponies, one German Riding pony, three Gotland Russ ponies, one Norwegian Fjord horse, three crossbred ponies, one Icelandic horse, 39 Shetland ponies, 23 Pura Raza Españolas (PRE) and three Puro Sangue Lusitanos (PSL) or mixed PRE/PSL horses, one Sorraia pony, one crossbred coldblood horse and one Yakut horse.

DNA was extracted from hair samples by cutting a ~ 1 cm long piece from the follicle end of ~ 25 hairs from each individual, then putting them into 200 µL of QuickExtract solution (Epicentre) and following the protocol of the manufacturer. For blood samples, DNA was extracted with the Mobio BloodKit according to the instructions of the manufacturer (Mobio).

### Mitochondrial analyses

We amplified a 774 bp long part of the mitochondrial control region by using the primers Eca_tRNAThr_L (5′-AAACCAGAAAAGGGGGAAAA-3′, [18]) and Eca_CR690_H (5′-TTGTTTCTTATGTCCCGCTACC-3′, designed for this study). PCR were carried out with 2 µL of 5 × Phusion buffer, 0.2 µL of 10 mM dNTPs, 0.5 µL of both primers (10 µM), 0.5 to 2 µL (~ 20 to 200 ng) of template DNA and 0.1 µL of Phusion DNA polymerase, the reaction conditions included an initial denaturation step at 98 °C for 30 s, followed by 35 cycles at 98 °C for 10 s, 53 °C for 30 s and 72 °C for 30 s, and a final extension for 10 min at 72 °C. PCR products were sequenced using the primer Eca_CR690_H, the BigDye Terminator v.3.1 kit and an ABI 3730 automatic sequencer (Applied Biosystems). Sequences were aligned by eye with the program BioEdit 7.2.5. [19]. In order to classify the sequences into previously defined haplogroups, we also included in the alignment the sequences from [11] (GenBank Accession Nos. JN398377–JN398457), and drew a haplotype network using TCS v. 1.21 [20]. When both a mare and its fowls were sampled, the fowls were excluded from the dataset.

We determined the best substitution model for the alignment with the program Mega 6.06 [21] and used the suggested model (see Results) to calculate pairwise Φ_ST_-values between the Finnhorse breeding sections with Arlequin v.3.5.1.3 [22]. Indices of DNA polymorphism (nucleotide diversity $\pi$, mutation parameter $\uptheta$, haplotype diversity ${\hat{\text{h}}}$ and number of haplotypes) were calculated with DnaSP v. 5.1 [23]. We also used the mitochondrial sequences to estimate the past and present female effective population sizes of the Finnhorse breed based on the Bayesian skyline plot in the program BEAST v.1.8.0 [24]. BEAUTI v.1.8.0 [24] was used to create an input file for BEAST, implementing the aforementioned substitution model with five gamma categories. We performed this analysis with two datasets, the first one including all the samples from the four Finnhorse breeding sections together with a random sample of 50 individuals that were not registered in the studbook, and the second one including only the samples of the breeding sections. Markov chain Monte Carlo (MCMC) was run for 10,000,000 steps, using ten groups and the piecewise-constant model. Posterior distributions and effective sample sizes were inspected with TRACER v.1.7.1 [25], which was also used to analyse the skyline. To transform the obtained times into years and female effective size estimates into individuals, we used the minimum mutation rate of 2.9 × 10^−6^ and maximum mutation rate of 10 × 10^−6^ [26].

### SNP data

We randomly sampled 12 horses from each breeding section of Finnhorses (trotters, riding horses, pony-sized horses and draught horses), 12 horses that were not in the studbook and 12 horses from other breeds, i.e. one Estonian horse, two Warmblood trotters, one Irish Cob, one Welsh Mountain, two Shetland ponies, one KWPN (Royal Dutch Sport horse), one Gotland Russ, one FWB (Finnish Warmblood), one Norwegian Fjord and one American Quarter horse (Table 1). These 72 horses were genotyped using the Illumina Equine SNP70 BeadChip at the laboratory of Dr. Van Haeringen (Wageningen, the Netherlands). This chip includes 65,157 SNPs across the horse genome. Across all samples, the average genotyping call rate was 0.988, after excluding three samples with call rates ranging from 0.355 to 0.419 (one mare and one stallion pony-sized Finnhorse and one mare Finnhorse that is not registered in the studbook; these individuals were not included in further analyses). The data were pruned for minor allele frequencies (MAF = 0.05) and linkage (with a sliding window of 50 SNPs, shifting the window 5 SNPs forward and removing SNPs with an r^2^ higher than 0.5; –maf 0.05 –indep-pairwise 50 5 0.5) using the PLINK 1.9 software [27, 28]. Finally, 37,445 SNPs remained for further analyses, unless stated otherwise.

**Table 1:** Samples analysed with the Illumina Equine SNP70 BeadChip, and the obtained expected heterozygosity and inbreeding estimates

Breed/breeding section	N mares	N stallions/geldings	H_E_	F_IS_	Mean number of ROH	Mean size of ROH (kb)	$\hat{F}_{\text{I}}$	$\hat{F}_{\text{II}}$	$\hat{F}_{\text{III}}$	F_PED_
Finnhorse/not in studbook	5	6	0.325	0.012	8.2	15,927	− 0.057	0.033	− 0.012	0.034
Finnhorse/trotter	6	6	0.318	0.004	9.5	13,854	− 0.067	0.046	− 0.010	0.045
Finnhorse/riding	5	7	0.324	0.010	8.2	13,978	− 0.049	0.035	− 0.007	0.039
Finnhorse/pony-sized	5	5	0.326	0.014	6.7	15,350	− 0.023	0.011	− 0.006	0.032
Finnhorse/draught	6	6	0.324	0.005	7.6	16,221	− 0.051	0.032	− 0.010	0.031
*All Finnhorses*	*27*	*30*	*0.324*	*0.016*	*8.1*	*15,036*	− *0.050*	*0.032*	− *0.009*	*0.036*
Estonian Horse	–	1	NA	NA	12	14,742	0.207	− 0.075	0.066	0.042
Warmblood trotter	1	1	NA	NA	32	10,674	0.588	− 0.012	0.288	0.131
Irish Cob	1	–	NA	NA	20	8994	0.290	0.043	0.166	NA
Welsh mountain	–	1	NA	NA	26	12,892	0.283	0.034	0.158	0.071
Shetland pony	1	1	NA	NA	21	10,162	0.279	0.114	0.196	0.020
KWPN (Royal Dutch Sport Horse)	1	–	NA	NA	11	8368	0.507	− 0.192	0.158	0.007
Gotland Russ	1	–	NA	NA	38	13,606	0.367	0.194	0.281	0.140
FWB (Finnish Warmblood)	1	–	NA	NA	13	6846	0.560	− 0.183	0.189	0.006
Norwegian Fjord	–	1	NA	NA	19	12,055	0.198	0.038	0.118	0.045
American Quarter	–	1	NA	NA	16	13,197	0.438	− 0.156	0.141	0.012
*Mixed breeds group*	*6*	*6*	*0.352*	*0.143*	*21.5*	*11,031*	*0.382*	− *0.008*	*0.187*	*0.057*

Note, if only one individual of a breed was analysed, the individual numbers and sizes of ROH and inbreeding coefficients are presented, but not averages

N = number of individuals, H_E_ = expected heterozygosity, F_IS_ = inbreeding coefficient based on difference between expected and observed heterozygosities, mean number of ROH = mean number of runs of homozygosity extending over 1000 kb, mean size of ROH extending over 1000 kb, $\hat{F}_{\text{I}}$ = inbreeding coefficient based on variance-standardized relationship minus 1, $\hat{F}_{\text{II}}$ = inbreeding coefficient based on excess of heterozygosity and $\hat{F}_{\text{III}}$ = inbreeding coefficient based on correlation between uniting gametes and F_PED_ = inbreeding coefficient based on pedigrees

### Genetic variation, inbreeding and effective population size

Genomic diversity and inbreeding were estimated with PLINK 1.9, using the functions –het (observed and expected homozygous genotype counts and method-of moments F coefficient), –ibc (inbreeding coefficients $\hat{F}_{\text{I}}$, which is the variance-standardized relationship minus 1, based on the variance of additive genetic values within an individual, $\hat{F}_{\text{II}}$, which is based on the excess of heterozygosity within an individual, and $\hat{F}_{\text{III}}$, which is based on the correlation between uniting gametes within an individual [29]) and –homozyg (runs of homozygosity, ROH, using a scanning window of 50 SNPs and recording a ROH when at least 100 SNPs are included across a total length of 1000 kb or more; the X chromosome was excluded). Pedigree-based inbreeding coefficients ($F_{\text{ped}}$) were obtained from the studbook provided by the Finnish trotting and breeding association [30] or calculated based on the pedigrees from a web portal of a pedigree database of horses that are registered mainly in Finland [31] with a minimum of six generations backwards. Observed and expected heterozygosities were calculated with Arlequin v.3.5.1.3 [22]. Correlations between the different estimates were calculated using the Pearson correlation coefficient. Past and present effective population sizes were estimated with SNeP v.1.1 [32], by setting default values, for horses of each breeding section, horses that were not in the studbook, all the Finnhorses and all data (including the mixed breed group). This program estimates the trends of the historical effective population size trajectories from SNP data based on linkage disequilibrium [32].

### Population genetic structure

First, we used a Bayesian iterative algorithm implemented in the program Structure v. 2.3.1 [33] to investigate the presence of population genetic structure by placing samples into groups formed by individuals sharing similar patterns of variation. We used all the 65,157 SNPs and set the length of burn-in periods at 1000, used 10,000 MCMC replications and set the number of populations (K) at 1 to 10 for 10 iterations and used the admixture model with correlated allele frequencies. We performed the runs without LOCPRIOR, i.e. a priori knowledge of the sampled breeding sections or breeds. The Structure results were used to calculate Evanno’s ΔK [34] in Excel. Furthermore, the iterations of the best number of populations (K = 2) were used to construct a barplot with the help of the output from the web-based program Structure Harvester v.0.6.94 [35], which was used as input for the program CLUMPP v.1.1.2 [36]. This program aligns the membership coefficients of the iterations. Then, barplots of the individual assignment probabilities were constructed with Distruct v.1.1 [37]. Population structuring was further studied with principal component analysis in PLINK 1.9, using the function –pca and by calculating pairwise *F*_ST_-values in Arlequin. Significance was estimated by 110 permutations.

## Results

### Genetic variation and inbreeding

Analysis of the mitochondrial sequences resulted in the alignment of a 631-bp long fragment from 743 Finnhorses and 121 horses of other breeds (fowls excluded). The best-fitting substitution model was TN93 + G + I, with a gamma shape parameter of 0.51 and proportion of invariant sites of 0.56 (BIC 34822.183 compared to the second best, GTR + G + I, 34843.342). Among all the horses, we detected 249 haplotypes and 158 polymorphic sites, with a nucleotide diversity of 0.022 (SD = 0.00016) and haplotype diversity of 0.982 (SD = 0.0015). Among the Finnhorses, we detected 203 haplotypes and 150 polymorphic sites, with a nucleotide diversity of 0.022 (SD = 0.00018) and haplotype diversity of 0.979 (SD = 0.0019). Among the horses from the four Finnhorse breeding sections, the diversity parameters showed little variation, with nucleotide diversities being highest for draught horses and lowest for trotters and haplotype diversity and θ being lowest for pony-sized horses (Table 2).

**Table 2:** Variation in mitochondrial control region sequence within the Finnhorse breeding sections

Breeding section	N	#	${\hat{\text{h}}}$	π	θ
Riding	76	46	0.978 (0.007)	0.0218 (0.00066)	0.0263 (0.00721)
Trotter	62	40	0.967 (0.012)	0.0216 (0.00086)	0.0275 (0.00778)
Draught	29	22	0.978 (0.015)	0.0224 (0.00142)	0.0287 (0.00941)
Pony-sized	46	28	0.965 (0.016)	0.0218 (0.00091)	0.0264 (0.00793)
Not in studbook	546	172	0.979 (0.0022)	0.0218 (0.00021)	0.0295 (0.00597)
All Finnhorses	*743*	*203*	*0.979 (0.0019)*	*0.022 (0.00018)*	*0.0333 (0.00644)*

Standard deviations are given in parentheses

N = number of individuals; # = number of haplotypes; ${\hat{\text{h}}}$ = haplotype diversity; π = nucleotide diversity and θ = mutation parameter from segregating sites

**Fig. 1:**
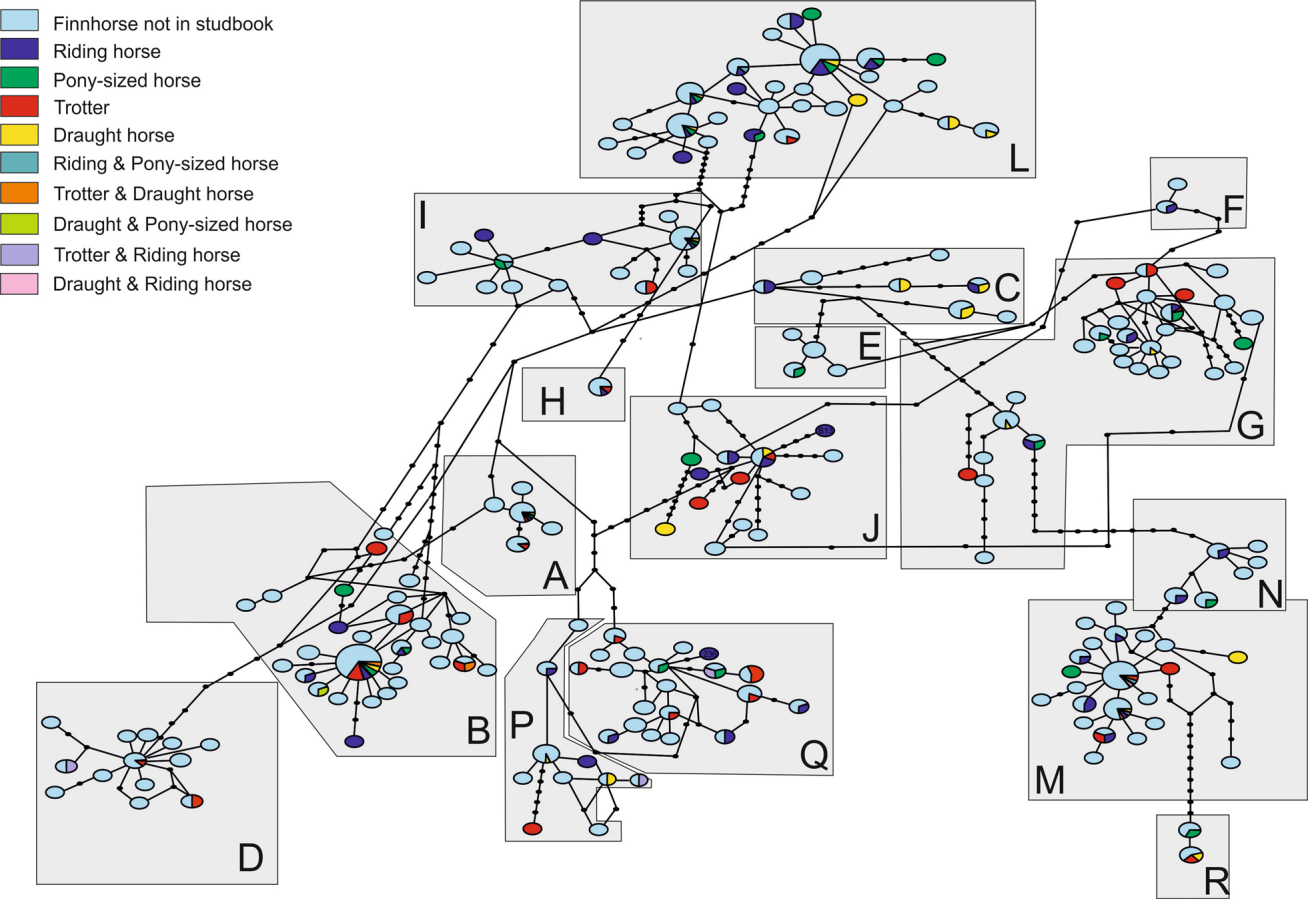
Haplotype network of the sequenced Finnhorses. Haplogroups are named after [11]

Of all haplogroups (A to R, as defined in [11]), all haplogroups except O and K were present in the Finnhorses (Fig. 1) and (see Additional file 1). The sequenced part of the control region seemed to perform as well as the whole mitogenome for assigning the sequences into haplogroups. In riding horses, the most frequent haplogroups were L and M, in trotters B and Q, in draught horses B, C and M, and in pony-sized horses G and L (Table 3). Interestingly, two of the haplotypes identified in the Finnhorses belong to haplogroup F, which is present in the Przewalski’s horse (*Equus przewalskii*). A Blast search against GenBank for these Finnhorse sequences also resulted in best matches with the Przewalski’s horse sequences (e: 0.0, identity: 626–627/630 bp). For the other breeds for which we had more than 20 representatives, Shetland ponies were in haplogroups D, G, I, L, M, N and Q, warmblood trotters in A, B, G, I, L and N and Pura Raza Españolas in B, G, L, N and Q (see Additional file 1).

**Table 3:** Haplogroup frequencies in the four Finnhorse breeding sections

Haplogroup	Riding N = 76	Trotter N = 62	Draught N = 29	Pony-sized N = 46	Not in studbook N = 546
A	0.013	0.032	0.035	0.022	0.035
B	0.079	*0.274*	*0.241*	0.130	*0.183*
C	0.040	0.000	*0.172*	0.000	0.020
D	0.026	0.081	0.000	0.044	0.022
E	0.000	0.000	0.000	0.022	0.015
F	0.013	0.000	0.000	0.000	0.005
G	0.053	0.081	0.069	*0.174*	0.115
H	0.013	0.016	0.000	0.000	0.009
I	0.053	0.032	0.000	0.087	0.055
J	0.066	0.048	0.069	0.022	0.024
K	0.000	0.000	0.000	0.000	0.000
L	*0.316*	0.097	0.103	*0.391*	*0.213*
M	*0.197*	0.129	*0.172*	0.044	0.148
N	0.026	0.000	0.000	0.022	0.020
O	0.000	0.000	0.000	0.000	0.000
P	0.040	0.032	0.035	0.000	0.040
Q	0.066	*0.161*	0.069	0.022	0.086
R	0.000	0.016	0.035	0.022	0.009

The two highest frequencies in each section are marked in italics. Haplogroup names A–R are after [9]

Within each Finnhorse breeding section, the values of expected heterozygosity from the SNP data were lowest for the trotters (0.318) and highest for the pony-sized horses (0.326). Average *F*_IS_, which was estimated with PLINK, ranged from 0.003 (pony-sized horses) to 0.027 (trotters). *F*_*IS*_-estimates from Arlequin were slightly different, ranging from 0.004 (trotters) to 0.014 (pony-sized horses) (see Table 1).

Compared to all other breeds, we found the smallest number of ROH in the Finnhorse breed, with an average number of 8.1 while it ranged from 11 to 38 in all the other breeds studied here (Table 1) and (see Additional file 2), but in Finnhorses, the ROH were longer (on average 15,036 kb), while they ranged from 6846 to 14,742 kb in the other breeds. The average distance spanned by ROH was 137.6 Mb in trotters, 117.8 Mb in riding horses, 104.4 Mb in pony-sized horses, 126.1 Mb in draught horses and 131.9 Mb in the mixed breed group. The largest numbers of ROH were found in a Gotland Russ (38, spanning 517.0 Mb) and in a Warmblood Trotter (34, spanning 362.9 Mb), whereas the smallest number (3) was found in three Finnhorse mares, each from a different breeding section, i.e. one trotter (spanning 25.8 Mb), one draught horse (spanning 35.5 Mb) and one riding horse (spanning 38.3 Mb). The smallest number of ROH in the other breeds (11) was found in a Royal Dutch Sport horse, spanning 92.0 Mb. The mean sizes of ROH in kb (extending the set 1000 kb size limit) are in Table 1. The three inbreeding coefficients estimated with PLINK and from pedigrees showed essentially the same pattern: Finnhorses were less inbred than the breeds in the mixed breed group (Finnhorses $\hat{F}_{\text{I}}$ = − 0.050, SD = 0.024, $\hat{F}_{\text{II}}$ = 0.032, SD = 0.028, $\hat{F}_{\text{III}}$ = − 0.009, SD = 0.015 and $F_{\text{ped}}$ = 0.036, SD = 0.015, mixed breeds $\hat{F}_{\text{I}}$ = 0.382, SD = 0.025, $\hat{F}_{\text{II}}$ = − 0.008, SD = 0.025, $\hat{F}_{\text{III}}$ = 0.187, SD = 0.025 and $F_{\text{ped}}$ = 0.057, SD = 0.056). For one horse (Irish Cob) of the mixed breed group, we could not find its pedigree. Inbreeding coefficients of individual Finnhorses varied less than those of the mixed breeds group ($\hat{F}_{\text{I}}$: from − 0.094 to 0.030 for Finnhorses and from 0.197 to 0.637 for the other breeds, $\hat{F}_{\text{II}}$: from − 0.020 to 0.079 for Finnhorses and from − 0.192 to 0.194 for the other breeds, $\hat{F}_{\text{III}}$: from − 0.038 to 0.033 for Finnhorses and from 0.066 to 0.334 for the other breeds, $F_{\text{ped}}$: from 0.011 to 0.075 for Finnhorses and from 0.000 to 0.166 for the other breeds). Inbreeding coefficients for each breeding section and breed are in Table 1. All correlations between the different inbreeding estimates were significant ($\hat{F}_{\text{I}}$ vs. $\hat{F}_{\text{II}}$, r = − 0.406 and p = 0.0005; $\hat{F}_{\text{I}}$ vs. $\hat{F}_{\text{III}}$, r = 0.948 and p < 0.00001; $\hat{F}_{\text{I}}$ vs. ROH, r = 0.745 and p < 0.00001; $\hat{F}_{\text{III}}$ vs. ROH, r = 0.878 and p < 0.00001; $\hat{F}_{\text{I}}$ vs. $F_{\text{ped}}$, r = 0.287 and p = 0.0168; $\hat{F}_{\text{II}}$ vs. $F_{\text{ped}}$, r = 0.380 and p = 0.0013; $\hat{F}_{\text{III}}$ vs. $F_{\text{ped}}$, r = 0.445 and p = 0.0001; ROH vs. $F_{\text{ped}}$, r = 0.622 and p < 0.00001), except between $\hat{F}_{\text{II}}$ and $\hat{F}_{\text{III}}$ (r = − 0.095 and p = 0.442) and between $\hat{F}_{\text{II}}$ and ROH (r = 0.191 and p = 0.117).

### Effective population sizes

**Fig. 2:**
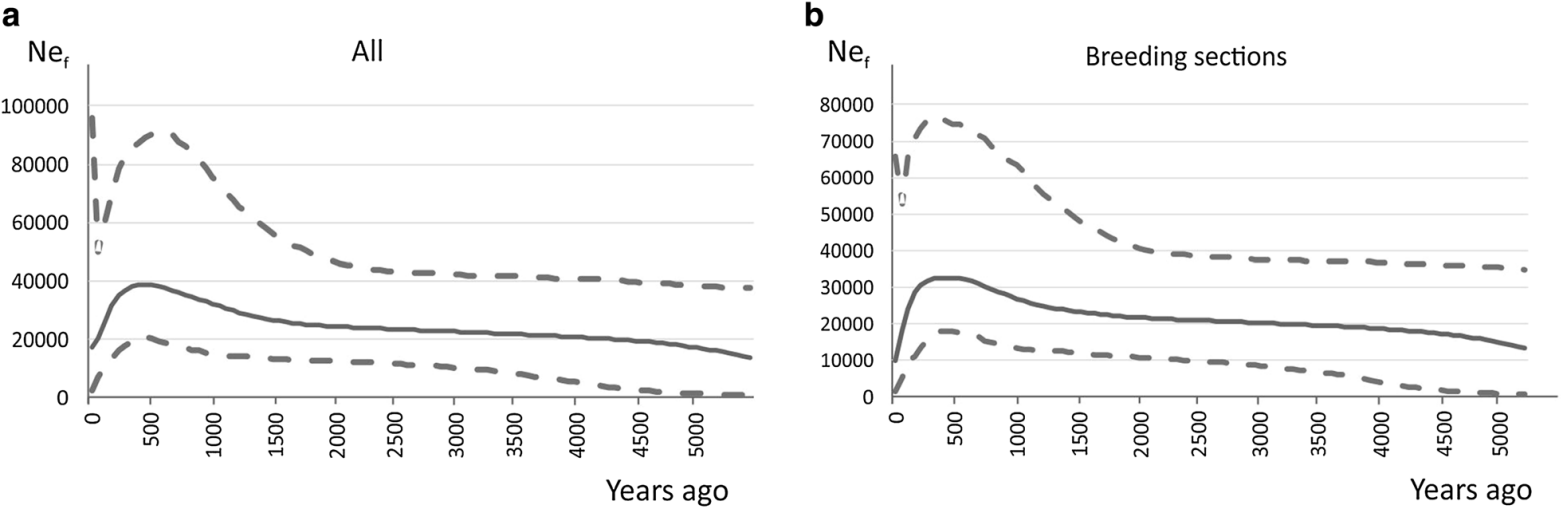
Skyline plots of the female effective population sizes of the Finnhorses based on a mutation rate of 2.9 × 10^−6^ **a** for all the horses of the four breeding sections and those not registered in the studbook horses combined together and **b** for only the horses of the four breeding sections

The Bayesian skyline results show that the female effective population size increased until approximately 110 to 300 years ago (applying the maximum and minimum mutation rates, respectively), at which point it started to decrease with a steep decline that started about 30 to 110 years ago (applying the maximum and minimum rates, respectively), both estimates including the time of foundation of the breed (Fig. 2a). When samples from only the Finnhorse breeding sections were analysed, the steep decline was more pronounced, and during the last 50 years, the female effective population size almost halved from 18,200 (8700 with the maximum rate) individuals to 9900 (2800 with the maximum rate) individuals (Fig. 2b). The current female effective population size of the Finnhorse breed that is estimated from the complete data, including the individuals that are not in the studbook, is equal to 17,200 (4900 with the maximum rate) individuals.

**Fig. 3:**
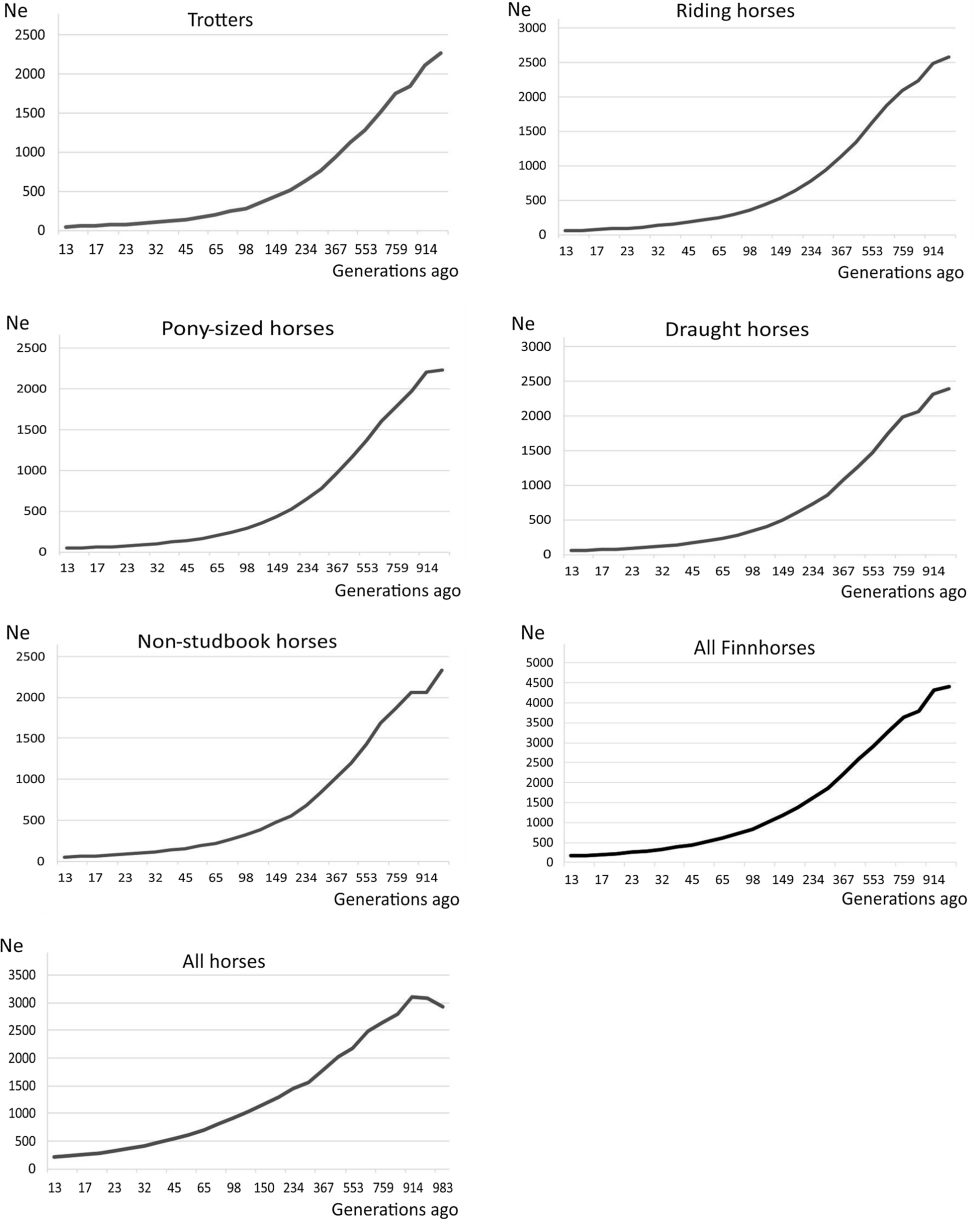
Effective population sizes estimated from SNP data

The effective population sizes estimated from the SNP data using SNeP were small, i.e. only 45 for trotters, 56 for riding horses, 43 for pony-sized horses, 52 for draught horses and 49 for horses that are not registered in the studbook. The effective population size for the entire Finnhorse data was equal to 161, and for the whole SNP data, i.e. including all the other breeds, 205. All the horses of the Finnhorse breeding sections as well as the horses that are not in the studbook showed a decline in effective population size over the complete estimated time period of 1000 generations. When the complete data was considered, the decline began slightly later, about 900 generations ago (Fig. 3).

### Genetic differentiation

**Fig. 4:**
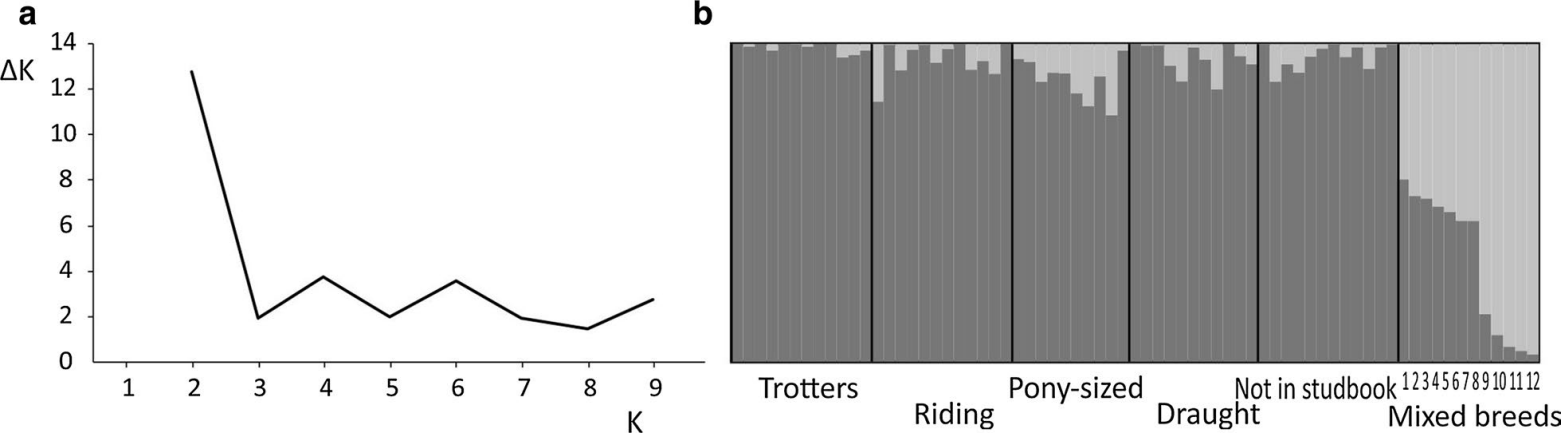
**a** Evanno’s ΔK values across different values for K (number of populations), of the genotyped horses. **b** Bar plot of the assignment probabilities for each individual at K = 2. Trotters, riding, pony-sized, draught and horses not registered in the studbook are Finnhorses, the numbered bars represent mixed breeds: 1 = Norwegian Fjord, 2 = Shetland pony, 3 = Gotland Russ, 4 = Shetland pony, 5 = Estonian horse, 6 = Irish Cob 7 = Welsh Mountain (section C), 8 = American Quarter, 9 = Dutch Warmblood, 10 = Finnish Warmblood, 11 and 12 = Warmblood Trotter

**Fig. 5:**
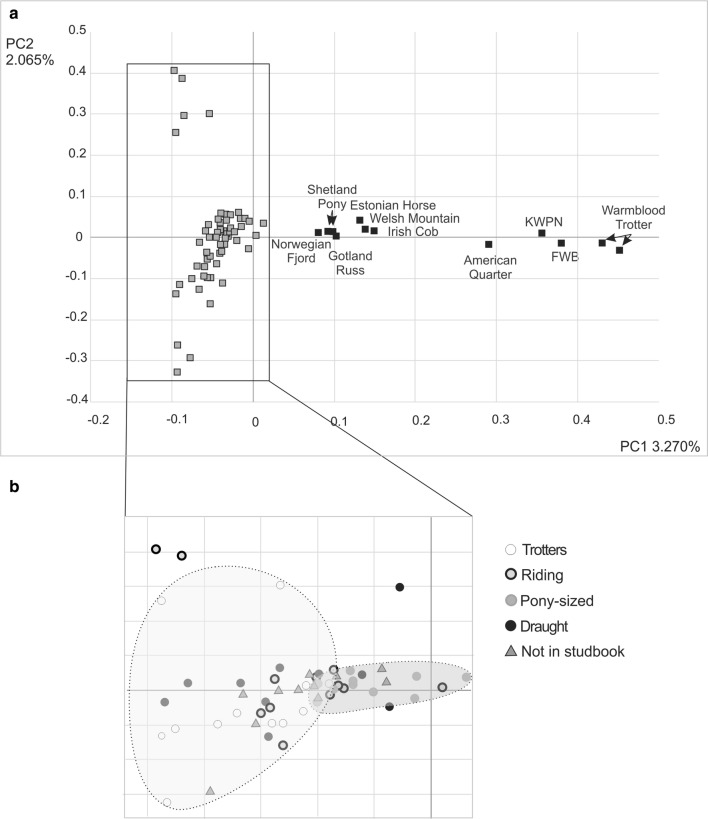
Principal component analysis of the genotyped horses. **a** All horses, with Finnhorses shown with grey squares and those of the other breeds with black squares. **b** Only Finnhorses with the individuals from the trotter and pony-sized breeding sections (encircled with light and dark grey, respectively) showing a slight clustering along PC1. Abbreviations: *KWPN* Royal Dutch Sport horse and *FWB* Finnish Warmblood

In the mitochondrial DNA dataset, the pairwise Φ_ST_-values were significant between riding horses versus trotters and draught horses, and between pony-sized horses *versus* trotters and draught horses. We detected no differentiation between trotters and draught horses or between pony-sized horses and riding horses (Table 4). For the SNP data, Evanno’s ΔK suggested that a value of 2 was the best number of genetic clusters, which separated the Finnhorses from the mixed breed group (Fig. 4). Similarly, the principal components analysis supported the separation between Finnhorses and the other breeds (Fig. 5a). Among the Finnhorse breeding sections, trotters and pony-sized horses are separated along the x-axis (Fig. 5b), but this is not clearly reflected by the *F*_ST_-values. The highest values were between the draught-type Finnhorses and all other groups, whereas the lowest values were between the riding and pony-sized Finnhorses (Table 4).

**Table 4:** Mitochondrial Φ_ST_-values between the Finnhorse breeding sections estimated with the Tamura and Nei substitution model (below the diagonal) and *F*_ST_-values for the SNP-genotyped horses (above the diagonal) between the Finnhorse breeding sections and a group of mixed breeds

	Trotter	Riding	Pony-sized	Draught	Non-studbook	Mixed breeds
Trotter		0.005	0.003	*0.018*	0.002	*0.045*
Riding	*0.054*		− 0.002	*0.009*	− 0.001	*0.035*
Pony-sized	*0.068*	0.015		*0.007*	− 0.001	*0.034*
Draught	− 0.005	*0.037*	*0.050*		*0.008*	*0.034*
Non-studbook	0.009	*0.015*	*0.024*	0.002		*0.035*
Mixed breeds	*0.050*	0.004	0.003	*0.038*	*0.015*	

Values in italics are significant at p < 0.05

## Discussion

### Genetic variation

Among our sample of 743 Finnhorses, we found 16 of the 18 previously described horse haplogroups, with only the European haplogroup K and the Middle Eastern haplogroup O missing. Interestingly, haplogroup F, which so far was detected only in the Przewalski’s horse, was present in four of the Finnhorses analysed here, of which three were not included in any of the breeding sections and one was registered as a riding horse. To date, haplogroup F has not been found in any modern horse breed, which is unexpected since it was recently suggested that the Przewalski’s horse, the only extant wild horse species, is derived from the early domestic horses of the Botai culture [38]. This result contradicted the findings of previous studies that have placed the Przewalski’s horse as a sister taxon of domestic horses, with some possible gene flow from the domestic horse to the Przewalski’s horse [39, 40]. The existence of the Przewalski haplogroup F in the Finnhorse, if verified by sequencing whole mitogenomes, may reveal surprising events in the domestication history of the horse.

Within the Finnhorse breeding sections, haplogroups L and M were most frequent in riding horses, haplogroups B and Q in trotters, haplogroups B, C and M in draught horses, and haplogroups G and L in pony-sized horses. All these haplogroups were considered as of European origin by [11] except for haplogroup Q, which has a more Asian or Middle Eastern distribution and is present, for example, in the Arabian horse. Mitochondrial DNA diversity in the Finnhorses ($\uppi$ = 0.022 and ${\hat{h}}$ = 0.979) is relatively similar to that detected in many other horse breeds [41–43]. In the domestic horse, it has been suggested that the large number of haplogroups and haplotypes spread over wide geographic regions results from a large number of mares having been incorporated into the domestic horse population [11, 12, 44]. This ancestral polymorphism has seemingly been retained in the Finnhorse as well, which is probably due to the high level of maternal genetic diversity of the founding population of the Finnhorse. Contrary to a previous Bayesian skyline plot that was constructed from a mixture of breeds in [11] and showed a decrease in the female effective population size about 7000 years ago and an increase thereafter, the female effective size in Finnhorses began to decrease approximately 300 years ago. This decline was accentuated approximately 110 years ago, at the time when the breed was founded, and again approximately 50 years ago, when the breeding sections for trotters, riding and pony-sized horses were founded (Fig. 2a, b). This is possibly due to a founder effect connected with the selection of horses that were included in the breed and in the breeding sections.

The present Finnhorse is closely related to the native Scandinavian, Estonian and Mongolian horses [6, 13] and presumably to the Russian heavy Mezen horse and the native Lithuanian Žemaitukas horse [14]. In our study, the Finnhorses also cluster with the native Estonian horse and with the native British and Irish breeds (Fig. 5). These close relationships between eastern, southern and western native breeds might explain the presence of haplotypes in the Finnhorse that are found in breeds from Europe, Central Asia and Middle East. Historically, King Gustav Vasa established stud farms in Finland as early as the 16^th^ century, with horses being imported from the Netherlands and northern Germany, to increase the size of Finnish horses. These horses most likely had the same ancestry as the modern Friesian and Oldenburg breeds, including the Spanish ancestry that was used to create the Friesian breed [45]. During and after the Thirty Years’ War (1618–1648), the Finnish cavalrymen who returned home brought with them horses from Central Europe and the Baltic region that were then bred with the Finnish horses. During the eighteenth and nineteenth centuries, some Arab horses were imported to Finland as well as Warmblood and heavy Ardennes horses from Sweden, and Orlov Trotters and ‘cossack horses’, possibly Don horses, from Russia [14, 46]. By the early twentieth century, all this crossbreeding had resulted in three different types of Finnish horses: heavy draught-type horses, light and long-legged (race) horses, and light and tough pony-sized horses [17]. Although most of the imported horses were stallions because they had more breeding value, occasionally imported mares may have introduced their Central European or eastern mitochondria into the Finnish horse population. Thus, the history of the breed may have resulted in a high level of mitochondrial genetic diversity and in a large female effective population size.

Analysis of the nuclear diversity, measured as the expected heterozygosity based on SNP data, showed that its level in Finnhorses was similar to that of many other breeds, including, for example, the Akhal-Teke, Andalusian, Lusitano, Mongolian and Tuva horses [6], which all have ‘moderate levels’ of nuclear diversity (the highest level i.e. 0.337 in Swiss Warmblood and Paint horses and the lowest level i.e. 0.239 in Clydesdale). The lowest and highest levels of diversity among our data for Finnhorse were in trotters (H_E_ = 0.318) and pony-sized horses (H_E_ = 0.326), respectively. These estimates are slightly higher than the estimates obtained from 27 Finnhorses (0.301) in [6], where the dataset was pruned for linkage equilibrium with an r^2^ > 0.4 compared to our r^2^ > 0.5, which might have had some effect on the estimates. Nevertheless, it is evident that the nuclear diversity of each of the Finnhorse breeding sections remains at a good level. Moreover, although ROH were longer in Finnhorses, there does not appear to be any strong inbreeding, since the numbers of ROH and inbreeding coefficients are smaller than in the other breeds that we studied here. Average inbreeding coefficients ($\hat{F}_{{\text{II}}}$) estimated in [6] varied from 0.015 in the Mongolian horse to 0.261 in Clydesdale, and was 0.052 for Finnhorses, whereas, based on a larger sample size, we found a lower estimate i.e. 0.032. Although the criteria used for the detection of ROH vary among studies, the length of the genome covered by ROH can be grossly compared between various studies of horse breeds. Using a 50-SNP window and setting the minimum length of ROH to 1000 kb, we found that the mean genome length covered by ROH in the Finnhorse breeding sections ranged from 104.4 to 137.6 Mb, whereas it ranged from 92.0 to 517.0 Mb in the other breeds that we studied. In a recent study [47], also based on a 50-SNP window but using another SNP chip to detect homozygous segments of more than 500 kb, the mean genome length covered by ROH was 305.1 Mb and ranged from 227.5 Mb in the Noriker breed (a heavy Austrian draught horse) to 396.5 Mb in Purebred Arabians. Another study [48], which used the previous version of the Illumina Equine BeadChip and a 50-SNP window to detect homozygous segments of more than 40 kb, found that the mean genome length covered by ROH ranged from 416.5 Mb in a Dülmen horse (a native German pony) to 953.2 Mb in a Thoroughbred.

Although we found no clear evidence of inbreeding, the effective population sizes for each breeding section and for the horses not registered in the studbook were very small (from 43 to 56) when estimated based on the nuclear SNPs. This small effective size can result from only having four founding stallions for the breed but may also stem from recent breeding practices; although such practices were designed to avoid inbreeding, only five stallions have made approximately 50% of the genetic contribution during the 2005–2014 period (estimated from pedigree data based on the expected proportion of alleles in an individual originating from an ancestor [49]). Currently, the number of matings for each stallion is limited to 150 per year [15], which could still be far too many if the aim is to increase the effective population size of the breeding sections and of the whole breed. Moreover, the overall number of stallions in each of the breeding sections is currently relatively small (218 stallions in harness trotters, 103 in riding horses, 64 in pony-sized horses and 30 in draught horses [15]), and the horses share many ancestors in their pedigrees. The effective population size of the entire breed obtained by combining all breeding sections and horses not in the studbook was 161, which is still a reasonably good level and might, in fact, favour the current breeding practice, which is that any registered Finnhorse is allowed to be included in the breeding sections provided that the section-criteria are met. Thus, the non-studbook horses serve as a large gene pool for the breeding sections.

### Genetic differentiation and history of breed

Based on the SNP data, we found a clear differentiation between the Finnhorses and the other breeds. All the Finnhorses clustered together in the Bayesian clustering analysis and along the first principal component in the PCA analysis, and were separated from the other breeds that we studied here. However, the differentiation between breeding sections was less clear. In the PCA, some clustering of the trotters and pony-sized horses was observed, but when we estimated *F*_ST_, *F*_ST_-estimates were significant only for the comparisons between the draught horses and the others (Table 4). Based on mitochondrial DNA, differentiation was strongest between the pony-sized horses and trotters, and overall, many pairwise comparisons were significant (Table 4). The only non-significant comparisons were between the trotters, draught horses and non-studbook horses and between the riding and pony-sized horses. The higher *F*_ST_-values obtained based on mitochondrial DNA compared to SNP data are most likely due to the different mode of inheritance of these two marker types: mitochondrial DNA is haploid and maternally inherited and thus has one-fourth of the effective population size of the diploid and biparentally inherited nuclear SNPs. This intensifies the effect of genetic drift on mitochondrial markers, leading to faster differentiation. The mainly non-significant *F*_ST_-values between the Finnhorse breeding sections suggest very weak, if any, differentiation among trotters, pony-sized horses and riding horses. The traits that are used as criteria for accepting individuals in the breeding sections are quantitative, possibly very complex and likely to have very variable heritabilities. Indeed, estimated heritabilities in the Finnhorse breed vary considerably from height at withers and at croup (0.89 and 0.90, respectively) to movement at walk and trot (0.13 and 0.18, respectively) [50]. The low heritabilities of many of the traits used, the acceptance of new individuals to breeding sections from the ‘common gene pool’ (i.e. non-studbook horses), the fact that several horses are registered in several breeding sections and that the breeding sections have been founded fairly recently, weaken the differentiation among the breeding sections.

## Conclusions

We found that the level of mitochondrial DNA variation was high in the Finnhorse, which confirms that the horse domestication in general involved a large number of mares, and the Finnhorse breed was founded using many local landraces. One of the haplogroups (F) that we identified in the Finnhorse breed has to date not been reported in domestic horses, but only in the Przewalski’s horse. This observation opens up new avenues for the study of the domestication of the horse. We show that the female effective population sizes in the Finnhorse and its breeding sections are large, but that their nuclear effective population sizes are small and the genetic differentiation between the breeding sections remains low. Although inbreeding in Finnhorses is lower than in many other horse breeds, the small nuclear effective population sizes can be considered a warning sign. The current common breeding practice of using only a few studs, which, e.g., have been successful in harness racing or riding competitions, should be restricted and the number of studs used should be increased. This could be achieved by, for example, further restricting the number of matings/stud and by encouraging the use of studs from rare pedigrees.

## Additional files

**Additional file 1.** Haplotype network of all the sequenced samples. White haplotypes (marked with an asterisk in the side panel) are from [11] and recovered from GenBank.

**Additional file 2.** Inbreeding estimates calculated from the genotyped horses. (a) $\hat{F}_{\text{I}}$, (b) $\hat{F}_{\text{II}}$, (c) $\hat{F}_{\text{III}}$, (d) mean number of runs of homozygosity, and (e) inbreeding coefficient estimated from pedigree data.

## Data Availability

All mitochondrial sequences are available in GenBank (Accession Nos. MN070243–MN071106). SNP data are available from the corresponding author upon request.
