# Supplementary material for: Genetic variability and history of a native Finnish horse breed
Source: Genet Sel Evol. 2019 Jul 1;51:35. doi: 10.1186/s12711-019-0480-8 (PMC6604459; doi:10.1186/s12711-019-0480-8)

Additional file 1. Haplotype network of all the sequenced samples. White haplotypes (marked with an asterisk in the sidepanel) are from [11] and fetched from GenBank.


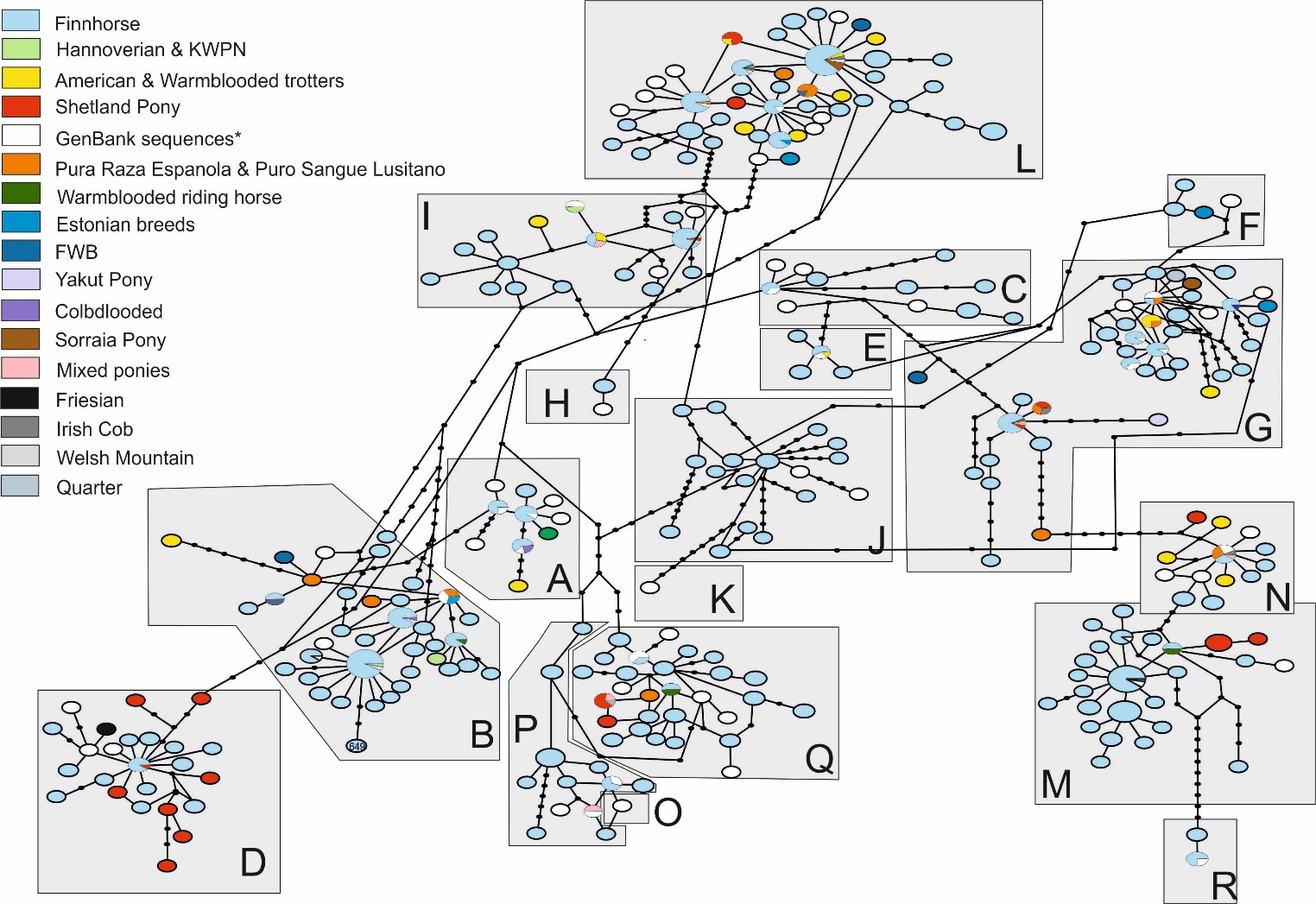

Supplement: Supplementary file 1 — Additional file 1. Haplotype network of all the sequenced samples. White haplotypes (marked with an asterisk in the side panel) are from [11] and recovered from GenBank. [file 12711_2019_480_MOESM1_ESM.docx]
