# Supplementary material for: Genetic variability and history of a native Finnish horse breed
Source: Genet Sel Evol. 2019 Jul 1;51:35. doi: 10.1186/s12711-019-0480-8 (PMC6604459; doi:10.1186/s12711-019-0480-8)

Additional file 2. Inbreeding estimates calculated from genotyped horses. a) $\hat{F}$_I_, b) $\hat{F}$_II_, c) $\hat{F}$_III_, d) mean number of runs of homozygosity and e) inbreeding coefficient estimated from pedigrees.


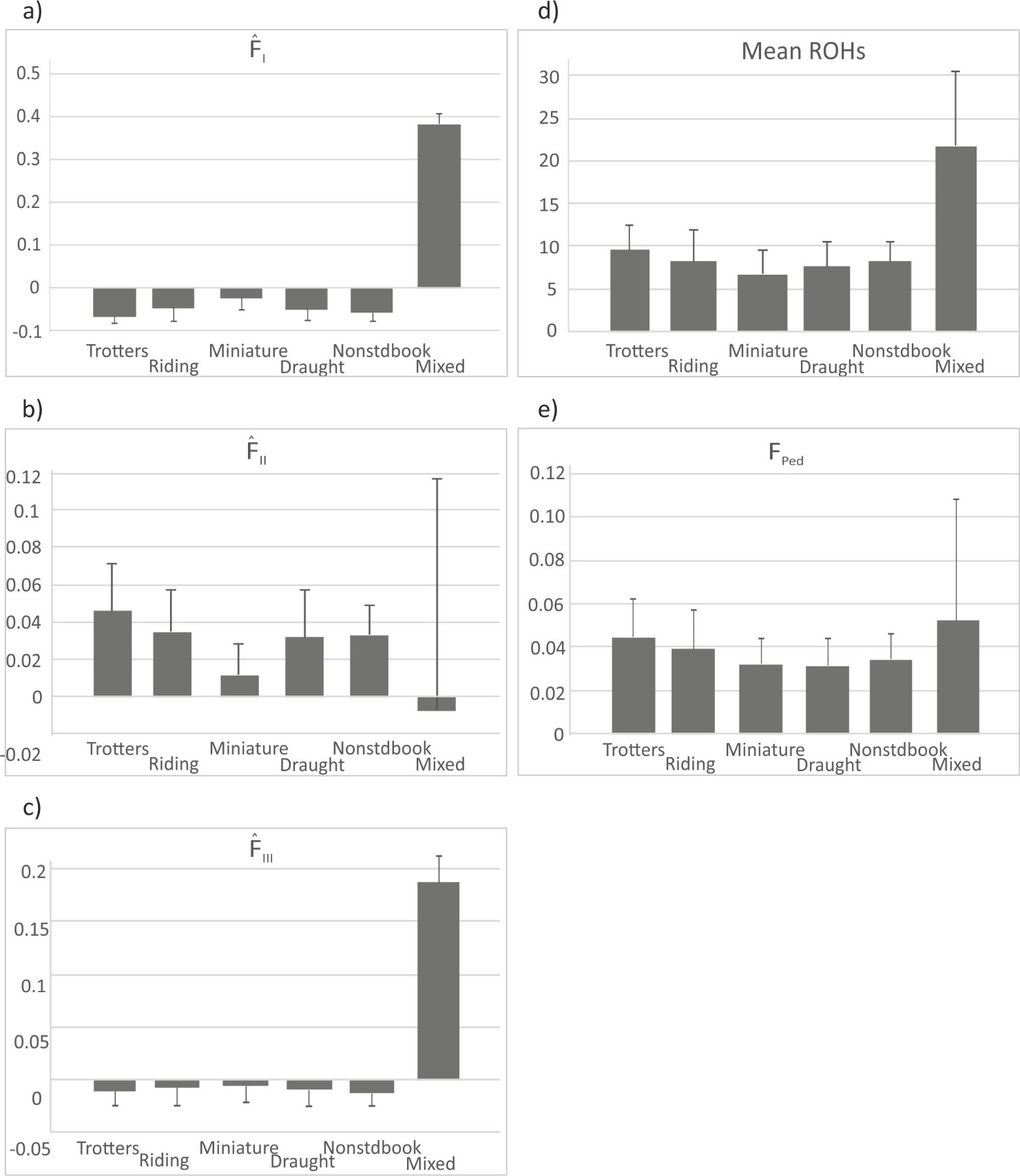

Supplement: Supplementary file 2 — Additional file 2. Inbreeding estimates calculated from the genotyped horses. (a) $\hat{F}_{\text{I}}$F^I, (b) $\hat{F}_{\text{II}}$F^II, (c) $\hat{F}_{\text{III}}$F^III, (d) mean number of runs of homozygosity, and (e) inbreeding coefficient estimated from pedigree data. [file 12711_2019_480_MOESM2_ESM.docx]
